# No evidence of a causal relationship between miscarriage and 25-hydroxyvitamin D: a Mendelian randomization study

**DOI:** 10.1093/hropen/hoae011

**Published:** 2024-02-19

**Authors:** Feng Zhang, Jingtao Huang, Gangting Zhang, Mengyang Dai, Tailang Yin, Chunyu Huang, Jue Liu, Yan Zhang

**Affiliations:** Reproductive Medical Center, Renmin Hospital of Wuhan University, Wuhan, Hubei, China; Department of Clinical Laboratory, Institute of Translational Medicine, Renmin Hospital of Wuhan University, Wuhan, Hubei, China; Reproductive Medical Center, Renmin Hospital of Wuhan University, Wuhan, Hubei, China; Wuhan Meizhao Health Management Co, Ltd, Wuhan, Hubei, China; Reproductive Medical Center, Renmin Hospital of Wuhan University, Wuhan, Hubei, China; Reproductive Medical Center, Renmin Hospital of Wuhan University, Wuhan, Hubei, China; Shenzhen Key Laboratory for Reproductive Immunology of Peri-implantation, Shenzhen Zhongshan Institute for Reproduction and Genetics, Shenzhen Zhongshan Obstetrics & Gynecology Hospital (formerly Shenzhen Zhongshan Urology Hospital), Shenzhen, Guangdong, China; Department of Epidemiology and Biostatistics, School of Public Health, Peking University, Beijing, China; Department of Clinical Laboratory, Institute of Translational Medicine, Renmin Hospital of Wuhan University, Wuhan, Hubei, China

**Keywords:** 25-hydroxyvitamin D, vitamin D deficiency, miscarriage, genome-wide association study, Mendelian randomization, causal effect

## Abstract

**STUDY QUESTION:**

Is there a causal relationship between 25-hydroxyvitamin D (25OHD) and miscarriage?

**SUMMARY ANSWER:**

In this study, little evidence of a causal relationship was found between low serum 25OHD concentration or vitamin D deficiency and the risk of miscarriages.

**WHAT IS KNOWN ALREADY:**

Associations between low vitamin D levels and increased risk of miscarriage have been reported, but causality is unclear.

**STUDY DESIGN, SIZE, DURATION:**

The latest and largest genome-wide association studies (GWAS) for serum 25OHD concentration (n = 417 580), vitamin D deficiency (426 cases and 354 812 controls), miscarriage (16 906 cases and 149 622 controls), and the number of miscarriages (n = 78 700) were used to explore the causal association between serum vitamin D levels and miscarriage by two-sample Mendelian randomization analysis.

**PARTICIPANTS/MATERIALS, SETTING, METHODS:**

This study was based on summary GWAS results from the FinnGen database and the UK Biobank. The random-effect inverse-variance weighted method was regarded as the primary analysis; MR-Egger, weighted median, weighted mode, simple mode, and MR-pleiotropy residual sum and outlier (MR-PRESSO) were further employed as complementary methods. MR-Egger intercept analysis and MR-PRESSO were employed to test pleiotropy, and Cochran’s Q statistic and leave-one-out sensitivity analysis were used to determine the heterogeneity and robustness of the overall estimates, respectively.

**MAIN RESULTS AND THE ROLE OF CHANCE:**

There was insufficient evidence of causal associations between serum 25OHD concentration and miscarriage (odds ratio (OR) = 0.995, 95% CI: 0.888 to 1.114, *P* = 0.927), or the number of miscarriages (β = –0.004, 95% CI: –0.040 to 0.032, *P* = 0.829). Furthermore, little evidence of causality between genetically determined vitamin D deficiency to miscarriage (OR = 0.993, 95% CI: 0.966 to 1.021, *P* = 0.624), or the number of miscarriages (β = 0.001, 95% CI: −0.009 to 0.011, *P* = 0.828), was observed. The results of the sensitivity analysis were robust, and no significant heterogeneity or horizontal pleiotropy was found.

**LIMITATIONS, REASONS FOR CAUTION:**

This study is limited by the absence of female-specific GWAS data and the limited amount of GWAS data available for this study, as well as the need for caution in generalizing the findings to non-European ethnic groups.

**WIDER IMPLICATIONS OF THE FINDINGS:**

These findings enhance the current understanding of the intricate association between vitamin D and pregnancy outcomes, challenging prevailing beliefs regarding the strong association with miscarriage. The results provide a special perspective that may prompt further exploration and potentially offer insights for guiding future research and informing clinical guidelines pertaining to the management of miscarriage.

**STUDY FUNDING/COMPETING INTEREST(S):**

This project was supported by the Hubei Provincial Natural Science Foundation Program General Surface Project (2022CFB200), the Key Research & Developmental Program of of Hubei Province (2022BCA042), the Fundamental Research Funds for the Central Universities (2042022gf0007, 2042022kf1210), and the Interdisciplinary Innovative Talents Foundation from Renmin Hospital of Wuhan University (JCRCWL-2022-001, JCRCYG-2022-009). All authors have no conflicts of interest to declare.

**TRIAL REGISTRATION NUMBER:**

N/A.

WHAT DOES THIS MEAN FOR PATIENTS? Vitamin D is crucial for overall health, particularly during pregnancy. Surprisingly, 57–83% of pregnant women worldwide have low levels of this essential vitamin. Low vitamin D levels have been linked to various pregnancy complications as well as miscarriage, which can be physically and emotionally distressing. While it is known that low vitamin D levels are associated with miscarriage, whether or not there is a direct cause-and-effect relationship remains unclear. We aimed to investigate this relationship using Mendelian randomization of genetic information based on several large European databases to remove interference from potential confounding factors. This study found that there is little evidence to suggest that low vitamin D levels directly cause miscarriage. In other words, it is important to maintain healthy vitamin D levels; it may not directly influence the risk of miscarriage. Given the vital role of vitamin D during pregnancy, we still recommend that women preparing for pregnancy and pregnant women ensure they have adequate vitamin D.

## Introduction

Vitamin D is an essential fat-soluble vitamin mainly synthesized by cholesterol in the skin in the presence of ultraviolet light, although it can also be taken in small amounts through food. Vitamin D is hydroxylated to 25-hydroxyvitamin D (25OHD) in the liver by 25-hydroxylase, and is eventually converted to 1,25OHD in the kidney by 1-α-hydroxylase to perform biological functions (Gallagher and Rosen, 2023). 25OHD is the major circulating form and has been recognized as a reliable indicator to evaluate the nutritional status of vitamin D. Vitamin D deficiency has become a common global health problem. According to the recommendations of the American Institute of Medicine, a serum 25OHD concentration below 10 ng/ml (25 nmol/l) was defined as vitamin D deficiency (Institute of Medicine (US) Committee to Review Dietary Reference Intakes for Vitamin D and Calcium, 2011), while the Endocrine Society’s guidelines provided more stringent criteria, considering a serum 25OHD concentration below 20 ng/ml (50 nmol/l) as vitamin D deficiency (Holick *et al.*, 2011). Both of these standards are widely utilized. Worldwide, 30 to 80% of individuals have serum 25OHD concentrations below 20 ng/ml, and 30 to 50% have concentrations below 10 ng/ml (Gallagher and Rosen, 2023). Pregnant women are at high risk for vitamin D deficiency; a meta-analysis reported the prevalence of pregnant women worldwide with 25OHD concentrations below 20ng/ml and 10 ng/ml were 64% and 9% in the Americas, 57% and 23% in Europe, 79% and 46% in the Eastern Mediterranean, 87% and N/A in South-East Asia, and 83% and 13% in the Western Pacific (Saraf et al., 2016).

Beyond maintaining the normal function of the skeletal–muscular system, vitamin D has been found to regulate some reproductive processes, such as extrachorionic trophoblast invasion, spiral artery remodeling, and crosstalk between immune cells and trophoblasts at the maternal–fetal interface (Chan *et al.*, 2015; Ganguly *et al.*, 2018; Kim *et al.*, 2018; Ji *et al.*, 2019; Zhang *et al.*, 2020; Xu *et al.*, 2023), which indicates the importance of vitamin D in pregnancy-related physiological and pathological processes. Additionally, a large number of clinical studies have focused on the association between maternal vitamin D status during preconception and pregnancy and adverse maternal and offspring outcomes in recent decades, and vitamin D deficiency has been found to be associated with miscarriage, preeclampsia, gestational diabetes mellitus, intrauterine growth restriction, low birth weight, preterm labor, stillbirth, and other pregnancy-related disorders (Heyden and Wimalawansa, 2018; Tamblyn *et al.*, 2022; Zhang *et al.*, 2022). Miscarriage, in particular, causes severe physical and psychological harm.

Miscarriage is defined as the loss of intrauterine pregnancy before 24 weeks gestation, with a complex etiological spectrum and notable clinical heterogeneity (Bender Atik *et al.*, 2018). Over 60% of early pregnancy losses are attributed to fetal chromosomal abnormalities, including trisomies, monosomies, and polyploidy (Alves et al., 2023), and are linked to advanced maternal and parental age (du Fossé *et al.*, 2020). Additionally, anatomical factors, infectious agents, immune dysregulation, endocrine factors, nutritional metabolic factors, and unhealthy lifestyle habits are believed to affect the chance of miscarriage (Alves et al., 2023; Zhang *et al.*, 2023a). Among these, vitamin D stands out as one of the most extensively researched nutrients in the context of miscarriage.

Previous studies have shown that women with recurrent miscarriage have a reduction in 25OHD levels and decreased expression of 1-α-hydroxylase and vitamin D receptors at the maternal–fetal interface (Wang *et al.*, 2016; Yan *et al.*, 2016; Li *et al.*, 2017). Multiple recent meta-analyses have also shown that pregnant women with vitamin D insufficiency/deficiency have a significantly higher risk of miscarriage and recurrent miscarriage (Chen *et al.*, 2022; Tamblyn *et al.*, 2022). However, there is a lack of high-quality evidence from randomized controlled studies. It is difficult to accurately evaluate the causal relationship from observational studies due to the interference from known and other unknown confounders. Mendelian randomization (MR) studies, which are increasingly being applied to explore causality within observational studies, may be a feasible solution to this problem.

MR is a type of instrumental variable (IV) analysis that utilizes single-nucleotide polymorphisms (SNPs) derived from genome-wide association studies (GWAS) as the IVs to explore and quantify causal relationships between exposures and outcomes. Since each individual’s SNPs are predetermined from birth and are not subject to change by external factors, MR could effectively avoid the confounding bias inherent in conventional epidemiologic studies (Burgess *et al.*, 2019; Skrivankova *et al.*, 2021). According to our current knowledge, there is a lack of MR studies to investigate the relationship between 25OHD and miscarriage. We hereby investigated the causal association between serum 25OHD concentration and miscarriage events using two-sample MR based on the latest GWAS summary results from the FinnGen database and the UK Biobank. Considering that the relationship between 25OHD and outcomes may be non-linear or that health effects only occur with actual vitamin D deficiency (Zhou and Hyppönen, 2023), we further explored the potential causal relationship between vitamin D deficiency and miscarriage.

## Methods

### Study design

We aimed to explore the causal association between serum vitamin D levels and miscarriage through two-sample MR. All procedures were strictly conducted following the MR reporting guidelines (STROBE-MR) (Skrivankova *et al.*, 2021). SNPs were selected as IVs, and SNPs were chosen to meet the following three assumptions: (i) strongly associated with the exposure; (ii) not related to any confounding factors related to both the exposure and the outcome; and (iii) not associated with the outcome, except through the exposure. Given the potential non-linear relationship between 25OHD and the outcome, we considered both serum 25OHD concentration (continuous variable) and vitamin D deficiency (categorical variable) as exposures. To enhance the reliability and stability of the results, we analyzed the causal relationships between exposure and both miscarriage (categorical variable) and the number of miscarriages (continuous variable).

### Data sources

The UK Biobank, a prospective cohort study with over 500 000 participants across the UK, recruited individuals aged 40 to 69 years during 2006 to 2010 (Sudlow *et al.*, 2015). FinnGen is a genomics and personalized medicine research project, a significant public–private partnership analyzing genome and health data from 500 000 Finnish biobank samples to explore the genetic basis of disease (Kurki *et al.*, 2023). We employed summary GWAS results based on the FinnGen database and the UK Biobank for our analysis. Details of the data are provided in Supplementary Table S1.

The GWAS data for serum 25OHD concentration came from 417 580 European participants in the UK Biobank, adjusted for their BMI (https://cnsgenomics.com/data/revez_20/Revezetal2020_25OHD_BMIcov.gz). A chemiluminescent immunoassay (Diasorin Liaison^®^) was employed to quantitatively determine 25OHD levels in blood samples, measuring the overall concentration of both 25OHD3 and 25OHD2. In the interpretation of MR analysis results for 25OHD, the MR estimates were elucidated in terms of the impact of a one-unit change in natural-log-transformed 25OHD levels, with a 1 SD alteration corresponding to 0.5 natural-log nmol/l. Despite the inclusion of both male and female participants, the authors observed no significant association between gender and 25OHD, affirming the suitability of these data for our analysis (Revez *et al.*, 2020). The GWAS data for vitamin D deficiency (426 individuals with vitamin D deficiency and 354 812 controls from an European population) were from the latest update of the FinnGen consortium R9 version (https://storage.googleapis.com/finngen-public-data-r9/summary_stats/finngen_R9_E4_VIT_D_DEF.gz), which combined genotype data from Finnish biobanks and digital health record data from Finnish health registries since the 1970s. The definition of vitamin D deficiency was established through physician-diagnosed information using the International Classification of Diseases (ICD) 8 to 10th codes, namely ICD-10 E55, ICD-9 268, and ICD-8 265. The MR estimates for vitamin D deficiency were reported as the impact per one-unit higher log-odds of vitamin D deficiency. The GWAS data on miscarriage were also sourced from the most recent FinnGen consortium R9 version. This database encompasses 16 906 individuals with a history of miscarriage and 149 622 controls, with a median age of 30.1 years, drawn from a Finnish population from the 1970s to the present (https://storage.googleapis.com/finngen-public-data-r9/summary_stats/finngen_R9_O15_ABORT_SPONTAN.gz). Miscarriage was defined using the ICD-10 O03, ICD-9 634, and ICD-8 643 codes. The GWAS data for the number of miscarriages were available from the MRC-IEU public database (GWAS ID: ukb-b-419), involving 78 700 European participants. Ethical approval was not required since all the included GWAS data were publicly available and had received prior approval from the respective ethical review boards.

### Selection and validation of SNPs

The aforementioned three assumptions were used to screen suitable SNPs. First, to ensure that SNPs chosen as IVs were strongly associated with the exposure, SNPs meeting a genome-wide significance threshold of *P* < 5e−8 were incorporated into this study. Additionally, linkage disequilibrium analysis did not exceed the specified limit (*r*^2^ < 0.001 within a clumping window of 10 000 kb). Only SNPs with SNP IDs in the exposure GWAS data were incorporated, without substituting proxy SNPs. The explained variance and potential weak instrument bias for each SNP were evaluated using *R*^2^ and *F*-statistics, respectively, with the *R*^2^ of each SNP being summed to obtain the overall *R*^2^. The formula is provided where *R*^2^ represents the proportion of variance explained by the SNP, EAF represents the effect allele frequency of the SNP, β represents the effect size of the SNP, and n represents the sample size of the GWAS. An *F*-statistic <10 indicates potential weak instrument bias and excludes corresponding SNPs (Chen *et al.*, 2019; Zhang *et al.*, 2023b).

$$R^{2}=2\times \left(1-\mathrm{EAF}\right)\times \mathrm{EAF}\times \beta^{2}$$

$$F=\frac{R^{2}}{1-R^{2}}\times \left(n-2\right)$$

Second, to ensure that the IV was independent of confounders related to both exposure and outcome, we utilized PhenoScanner to validate the independence of all SNPs obtained from the previous step, removing those associated with confounders (http://www.phenoscanner.medschl.cam.ac.uk/) (Kamat *et al.*, 2019). Third, to ensure that the IV affected the outcome only through the exposure, we conducted a pleiotropy test to identify and remove SNPs exhibiting horizontal pleiotropy.

### Statistical analysis

The random-effect inverse-variance weighted (IVW) method was regarded as the primary analysis (Burgess *et al.*, 2019), and MR-Egger, weighted median, weighted mode, simple mode, and MR-pleiotropy residual sum and outlier (MR-PRESSO) were further employed as complementary methods (Zhang *et al.*, 2023b). MR-Egger intercept analysis and MR-PRESSO were employed to test horizontal pleiotropy and then outlier SNPs reflecting pleiotropic biases (Bowden *et al.*, 2015; Verbanck *et al.*, 2018). Additionally, Cochran’s Q statistic was used to test heterogeneity among selected SNPs, and a leave-one-out sensitivity analysis was conducted to determine the robustness of overall estimates by omitting individual SNPs one by one. A Bonferroni-corrected threshold of *P *<* *0.025 (α = 0.05/2 outcomes) was utilized to account for multiple testing in each exposure on two separate outcomes. All statistical analyses were performed using the ‘TwoSampleMR’ packages (version 0.5.6) and ‘MRPRESSO’ packages (version 1.0) in R (version 4.2.1; R Foundation for Statistical Computing, Vienna, Austria).

## Results

### SNPs selection and validation

**Figure 1.**
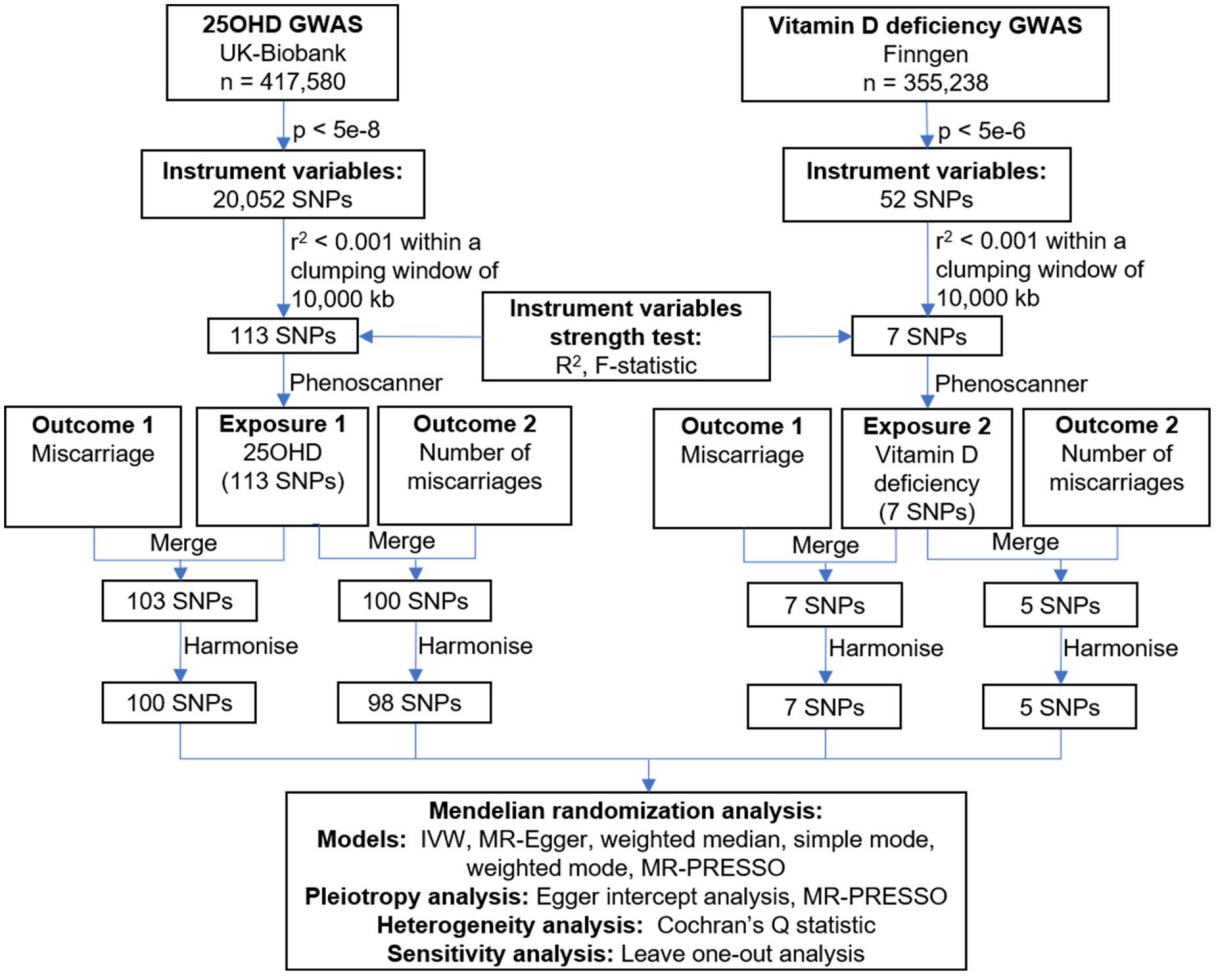
**Overview of data sources and the process of single-nucleotide polymorphisms selection and four independent two-sample Mendelian randomization analyses.** 25OHD: 25-hydroxyvitamin D; GWAS: genome-wide association studies; IVW: inverse-variance weighted; MR-PRESSO: MR-pleiotropy residual sum and outlier; SNPs: single-nucleotide polymorphisms.

The data for this study were sourced entirely from the UK Biobank and the FinnGen database, based on the European population (Supplementary Table S1). The process of SNPs selection and validation is shown in Fig. 1. Regarding the serum 25OHD concentration, after the removal of linkage disequilibrium, a total of 113 SNPs reached genome-wide significance (*P* < 5e−8), and all *F*-statistics were >10, with an explained variance of *R*^2^ = 2.8%. Ultimately, 100 and 98 SNPs were used to explore the causal relationship between serum 25OHD concentration and the odds and number of miscarriages, respectively (Supplementary Table S2). For vitamin D deficiency, no effective SNPs were extracted when setting *P* < 5e−8 or *P* < 5e−7. Therefore, the threshold was relaxed to *P* < 5e−6, resulting in seven SNPs. The *F*-statistics test indicated no weak IVs and a high explained variance of 34.6%. Eventually, seven and five SNPs were, respectively, used to explore the causal relationship between vitamin D deficiency and the odds and number of miscarriages (Supplementary Table S3).

### Serum 25OHD concentration and miscarriage

**Figure 2.**
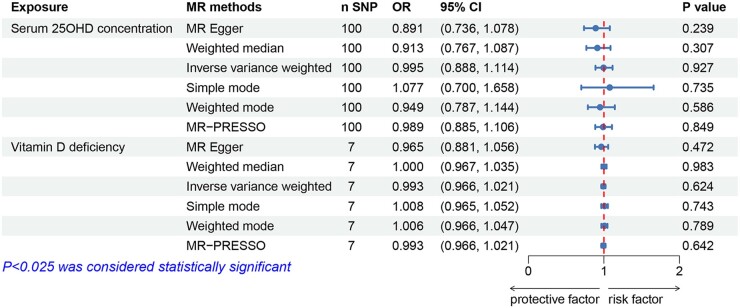
**Effects of genetically determined serum 25-hydroxyvitamin D concentration and vitamin D deficiency on the odds of miscarriage.** 25OHD: 25-hydroxyvitamin D; MR: Mendelian randomization; MR-PRESSO: MR-pleiotropy residual sum and outlier; OR: odds ratio; SNPs: single-nucleotide polymorphisms.

**Figure 3.**
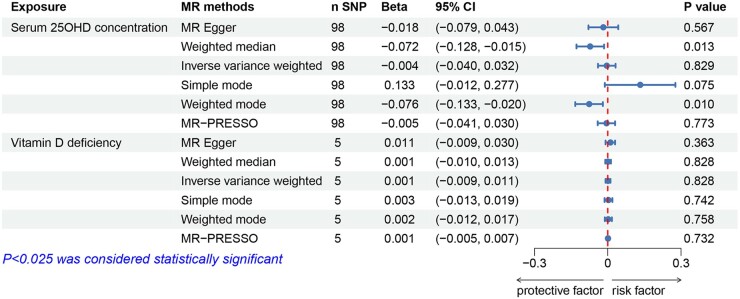
**Effects of genetically determined serum 25-hydroxyvitamin D concentration and vitamin D deficiency on the number of miscarriages.** 25OHD: 25-hydroxyvitamin D; MR: Mendelian randomization; MR-PRESSO: MR-pleiotropy residual sum and outlier; SNPs: single-nucleotide polymorphisms.

The primary IVW analysis indicated that for each one-unit increase in genetically determined natural-log-transformed serum 25OHD concentration, there was little causal association with decreased odds of miscarriage (odds ratio (OR) = 0.995, 95% CI: 0.888 to 1.114, *P* = 0.927; Fig. 2), nor with the number of miscarriages (β = –0.004, 95% CI: –0.040 to 0.032, *P* = 0.829; Fig. 3). Except for MR analysis of the 25OHD on the number of miscarriages in weighted median and weighted mode, the results from other MR analysis methods were consistent with the primary IVW results (all *P* > 0.025). Scatter plots and forest plots of the association between serum 25OHD concentration and miscarriage showed similar results (Supplementary Figs S1A and B and S2A and B). After sequentially excluding selected SNPs, all MR results crossed the null line, confirming result stability and suggesting minor heterogeneity among the SNPs (Supplementary Fig. S3A and B). Funnel plots were symmetrical, indicating no evidence of selection bias (Supplementary Fig. S4A and B). As shown in Table 1, Cochran’s Q statistics did not indicate significant heterogeneity among the SNPs of 25OHD, Egger intercept analysis detected no horizontal pleiotropy, and no outlier SNPs were observed in the MR-PRESSO analysis.

**Table 1.** Pleiotropy and heterogeneity test for Mendelian randomization (MR) analysis.

Exposure	Outcome	Pleiotropy analysis					Heterogeneity analysis					
		Egger intercept analysis			MR-PRESSO		Inverse-variance weighted			MR-Egger		
		Egger intercept	SE	*P*-value	RSSobs	*P*-value	Q	DF	*P*-value	Q	DF	*P*-value
25OHD	Miscarriage	0.004	0.003	0.164	114.650	0.226	109.731	99	0.217	107.575	98	0.239
	Number of miscarriages	0.0005	0.001	0.580	122.639	0.082	115.833	97	0.093	115.461	96	0.086
Vitamin D deficiency	Miscarriage	0.014	0.021	0.537	7.258	0.478	6.092	6	0.413	5.601	5	0.347
	Number of miscarriages	–0.006	0.006	0.350	2.324	0.821	1.402	4	0.844	0.185	3	0.980

25OHD: 25-hydroxyvitamin D; DF, degree of freedom; MR-PRESSO: MR-pleiotropy residual sum and outlier; Q, Cochran’s Q statistic; RSSobs: observed residual sum of squares.

### Vitamin D deficiency and miscarriage

Similarly, a one-unit increase in the log-odds of genetically determined vitamin D deficiency showed little causal association with increased odds of miscarriage (OR = 0.993, 95% CI: 0.966 to 1.021, *P* = 0.624; Fig. 2), nor the number of miscarriages (β = 0.001, 95% CI: –0.009 to 0.011, *P* = 0.828; Fig. 3). The results from other MR analysis methods aligned with the primary IVW findings (all *P* > 0.025). Scatter plots and forest plots of the association between vitamin D deficiency and miscarriage demonstrated consistent findings (Supplementary Figs S1C and D and S2C and D). After sequentially excluding the selected SNPs, all MR results did not change significantly, demonstrating the reliability of the results (Supplementary Fig. S3C and D). Cochran’s Q statistics did not display significant heterogeneity among the SNPs of vitamin D deficiency, and both Egger intercept analysis and MR-PRESSO analysis detected no notable horizontal pleiotropy (Table 1).

## Discussion

To our knowledge, this was the first study to use two-sample MR to explore the potential causal relationship between 25OHD and miscarriage. This MR analysis was based on robust IVs from large-sample GWAS databases of the European population. To enhance the accuracy of the results, we selected serum 25OHD concentration and vitamin D deficiency as exposures, and selected miscarriage and its number as outcomes, conducting four sets of two-sample MR analyses. In conclusion, our research found little evidence to support a causal association between genetically determined serum 25OHD concentration or vitamin D deficiency and the odds or number of miscarriages. These findings contribute to the current understanding of the complex relationship between vitamin D and pregnancy outcomes.

Given the crucial physiological significance of vitamin D in reproductive processes and the high prevalence of vitamin D deficiency among expectant mothers, the association between vitamin D levels and miscarriage has emerged as a prominent subject of research, warranting significant attention. Most observational studies have suggested an association between low levels of vitamin D and an increased risk of miscarriage. For instance, Tamblyn *et al.* (2022) conducted a systematic review and meta-analysis and found that vitamin D deficiency and insufficiency were associated with miscarriage. Similarly, Chen *et al.* (2022) found that patients with recurrent miscarriage had significantly lower serum 25OHD concentrations than normal pregnant women, and vitamin D deficiency during pregnancy might be a high risk factor for recurrent miscarriage. However, some studies did not find this association (Subramanian *et al.*, 2022). Moreover, clinical evidence from various RCTs indicated that vitamin D supplementation did not improve miscarriage rates as expected (Tamblyn *et al.*, 2022; Zhou *et al.*, 2022; Meng *et al.*, 2023). Although abundant exposure to ultraviolet radiation during the summer leads to a significant increase in serum vitamin D levels, research has not found any evidence suggesting improved clinical pregnancy rates, live birth rates, or reduced miscarriage rates in women undergoing oocyte retrieval and embryo transfer during the summer (Carlsson Humla *et al.*, 2022). These controversial findings prompted us to investigate the causal relationship between vitamin D and miscarriage, rather than the mere association. The causal relationship between miscarriage and vitamin D deficiency remains inconclusive due to the limitations of observational studies and the lack of high-quality randomized controlled trials. Our MR study provides new insights and offers an essential perspective on this debated topic. Although the observational findings suggested an association, a causal relationship between serum 25OHD concentration, vitamin D deficiency, and miscarriage was not supported by our MR analysis. This discrepancy could be attributed to confounding factors or biases inherent in observational studies, such as maternal age, BMI, smoking and alcohol history, socioeconomic status, chronic diseases, sampling season, and other unknown confounders, which are minimized in MR analysis (Veleva *et al.*, 2008; Pereira-Santos *et al.*, 2015; Pourshahidi, 2015; Quenby *et al.*, 2021; Lin *et al.*, 2022a, 2022b).

Although the results of this article and current clinical research did not support the prevention of miscarriage through vitamin D supplementation before and during pregnancy (Tamblyn *et al.*, 2022; Zhou *et al.*, 2022; Meng *et al.*, 2023), we still advocate for the supplementation of vitamin D in women preparing for pregnancy and in pregnant women with vitamin D insufficiency or deficiency in achieving normal levels of serum 25OHD. Pregnant women have an increased demand for vitamin D during pregnancy. Vitamin D supplementation not only meets the normal physiological needs of the mother, but also contributes to the development of the fetal musculoskeletal system, as the necessary vitamin D for the fetus is exclusively transferred through the placenta (Karras *et al.*, 2018; Kiely *et al.*, 2020). The vitamin D nutritional status of infants is closely linked to that of their mothers. Newborns from mothers with untreated vitamin D deficiency are more prone to vitamin D deficiency compared to infants born to mothers who have received vitamin D supplementation. Vitamin D deficiency in infants may have adverse effects on innate immune function and skeletal development (Dawodu and Wagner, 2007). Furthermore, studies have indicated that vitamin D supplementation could reduce the occurrence of adverse maternal and fetal events such as preeclampsia, gestational diabetes mellitus, low birth weight, and preterm delivery (Rostami *et al.*, 2018; Tamblyn *et al.*, 2022). Additionally, some studies have suggested potential benefits of vitamin D supplementation in enhancing clinical pregnancy rates for infertile women (Meng *et al.*, 2023).

Hence, the question of how to appropriately supplement vitamin D has emerged as a significant scientific concern. Vitamin D is primarily synthesized in the skin from cholesterol upon exposure to ultraviolet light, with dietary intake contributing only 10% of the body’s requirements (Holick and Chen, 2008; Heyden and Wimalawansa, 2018). Consequently, it is advocated that pregnant women with vitamin D deficiency receive more sun exposure, as it is a cost-effective and efficacious strategy. If, geographical factors, nature of work, lifestyle habits, darker skin tone, or other reasons result in insufficient exposure to ultraviolet light, or if there is an enzyme deficiency causing a synthesis obstacle for vitamin D, oral vitamin D supplements can be a good choice. The nutritional status of vitamin D is greatly influenced by latitude and race. Therefore, there are currently no unified guidelines for the use of vitamin D supplements. Different countries or regions have successively issued clinical guidelines that suit their local conditions (Hanley et al., 2010; Holick *et al.*, 2011; Institute of Medicine (US) Committee to Review Dietary Reference Intakes for Vitamin D and Calcium, 2011; WHO Guidelines Approved by the Guidelines Review Committee, 2012; Haq *et al.*, 2018; Pludowski *et al.*, 2018; Gupta *et al.*, 2022; Płudowski *et al.*, 2023).

While MR studies are able to mitigate the adverse effects of confounders and investigate the causal relationship between exposures and outcomes, this study has several limitations. First, the GWAS database from which the 25OHD concentration and vitamin D deficiency were derived included both males and females. This might affect the accuracy of the results, yet there currently exists no female-specific GWAS data. Nevertheless, the authors found no significant association between gender and 25OHD, indicating that vitamin D levels were not influenced by gender, and the data remained competent for analysis in this study (Revez *et al.*, 2020). Second, as no valid SNPs could be identified for vitamin D deficiency at the thresholds of *P* < 5e−8 or *P* < 5e−7, the threshold was subsequently adjusted to *P* < 5e−6. However, even at this relaxed threshold, the *F*-statistic test detected no weak IV and accounted for 34.6% of the observed variance. Third, the participants in this study all hail from European populations; therefore, caution should be exercised when extrapolating our conclusions to other ethnic groups. Currently, the majority of GWAS studies worldwide originate from European populations. However, there is a growing trend toward GWAS studies involving Asian and other ethnic groups. We look forward to the prompt release of GWAS data from diverse populations, aiming to comprehensively address this important issue. Fourth, due to the unavailability of relevant SNPs, we could not further explore the causal relationship between vitamin D supplementation and miscarriage, which holds immense clinical significance. Similarly, we could not locate any GWAS data related to recurrent miscarriage, preventing us from discussing the relationship between vitamin D and recurrent miscarriage. Fifth, many large-scale GWAS draw data from the UK Biobank and FinnGen datasets. The genetic IVs in our study were derived from these datasets, posing a risk of sample overlap in our MR analysis. Two-sample MR analysis assumes no sample overlap between exposure and outcome GWAS, with the expected covariance of sampling error between genetic effects on exposure and outcome presumed to be zero. Violating this assumption may lead to an inflated type I error rate in hypothesis testing for causal effects. However, as our MR results were entirely null, any potential inflation in the type I error rate does not impact the interpretation of our findings (Burgess *et al.*, 2016; Jiang *et al.*, 2021). Sixth, in many MR investigations involving binary exposures, particularly when the binary variable is a dichotomization of continuous exposure, there exists an underlying continuous risk factor (Burgess and Labrecque, 2018). In other words, variations in the SNPs may lead to alteration in 25OHD and consequently to changes in the outcome even if the exposure status for vitamin D deficiency remains fixed. Even so, we have already confirmed the absence of a significant causal relationship between continuous 25OHD and the outcomes in this study. Therefore, this risk may not substantially affect the MR analysis of vitamin D deficiency. Seventh, the definitions of miscarriage and vitamin D deficiency are not consistent across different periods. In this study, the GWAS data from the UK Biobank and FinnGen included multiple versions of ICD codes for defining these two objects, but specific definitions were not provided, potentially introducing intra-group heterogeneity in the data. Furthermore, these two definitions were based on doctors’ diagnosis, which may pose a risk of possible underdiagnosis for some individuals. Specifically, early miscarriages may be mistaken for menstrual periods leading to underdiagnosis, and the lower prevalence of vitamin D deficiency (0.12%) in the study population also suggests the possibility of underdiagnosis. This may be a common issue in GWAS for phenotypes defined by doctors’ diagnosis. Eighth, while MR analysis has evident advantages in elucidating causal relationships between exposures and outcomes, it is important to note that the genetic variation explained by SNPs represents only a portion of the overall exposure variance. Therefore, the MR model cannot capture the effect of exposure variance on outcome determined by other non-genetic factors. Last but not least, considering that the majority of miscarriages are attributed to fetal chromosomal abnormalities, and maternal vitamin D levels seem to have no significant association with fetal chromosomal abnormalities, in addition to anatomical factors, endocrine factors, immune dysregulation, infectious agents, and other risk factors. This implies that even if there exists a causal relationship between miscarriage and maternal serum vitamin D levels, it may only represent a very small fraction of cases. However, in our MR study, the data from the miscarriage population encompassed the entire spectrum of etiologies, potentially underestimating the causal relationship between vitamin D levels and miscarriage.

In summary, due to the aforementioned limitations, the findings of this study should be interpreted with caution. Further validation of our results is needed through large-scale randomized controlled studies in the future. Our team will continue to focus on this issue, anticipating the release of gender-specific, larger sample size, and ethnically diverse GWAS data to design more rational MR studies exploring the causal relationship between vitamin D and miscarriage.

## Conclusion

This two-sample MR study found little evidence to support a causal association between genetically determined serum 25OHD concentration or vitamin D deficiency and the odds or number of miscarriages in European women. However, the findings should be interpreted cautiously due to certain limitations. Future GWAS data specifically for women of childbearing age, as well as for other ethnic groups, are needed to further assess whether there is a causal association between 25OHD levels and miscarriage.

## Supplementary Material

hoae011_Supplementary_Data

## Data Availability

GWAS summary statistics analyzed in this study are publicly available and can be found at: serum 25-hydroxyvitamin D concentration: https://cnsgenomics.com/data/revez_20/Revezetal2020_25OHD_BMIcov.gz; vitamin D deficiency: https://storage.googleapis.com/finngen-public-data-r9/summary_stats/finngen_R9_E4_VIT_D_DEF.gz; miscarriage: https://storage.googleapis.com/finngen-public-data-r9/summary_stats/finngen_R9_O15_ABORT_SPONTAN.gz; and number of miscarriages: https://gwas.mrcieu.ac.uk/datasets/ukb-b-419/.

## References

[hoae011-B1] Alves C , Jenkins SM, Rapp A. *Early Pregnancy Loss (Spontaneous Abortion). StatPearls*. Treasure Island, FL: StatPearls Publishing, 2023. http://www.ncbi.nlm.nih.gov/books/NBK560521/ (12 October 2023, date last accessed). 32809356

[hoae011-B2] Bender Atik R , Christiansen OB, Elson J, Kolte AM, Lewis S, Middeldorp S, Nelen W, Peramo B, Quenby S, Vermeulen N et al ESHRE guideline: recurrent pregnancy loss. Hum Reprod Open 2018;2018:hoy004. 31486805 10.1093/hropen/hoy004 PMC6276652

[hoae011-B3] Bowden J , Davey Smith G, Burgess S. Mendelian randomization with invalid instruments: effect estimation and bias detection through Egger regression. Int J Epidemiol 2015;44:512–525. 26050253 10.1093/ije/dyv080 PMC4469799

[hoae011-B4] Burgess S , Davey Smith G, Davies NM, Dudbridge F, Gill D, Glymour MM, Hartwig FP, Holmes MV, Minelli C, Relton CL et al Guidelines for performing Mendelian randomization investigations. Wellcome Open Res 2019;4:186. 32760811 10.12688/wellcomeopenres.15555.1 PMC7384151

[hoae011-B5] Burgess S , Davies NM, Thompson SG. Bias due to participant overlap in two-sample Mendelian randomization. Genet Epidemiol 2016;40:597–608. 27625185 10.1002/gepi.21998 PMC5082560

[hoae011-B6] Burgess S , Labrecque JA. Mendelian randomization with a binary exposure variable: interpretation and presentation of causal estimates. Eur J Epidemiol 2018;33:947–952. 30039250 10.1007/s10654-018-0424-6 PMC6153517

[hoae011-B7] Carlsson Humla E , Bergh C, Akouri R, Tsiartas P. Summer is not associated with higher live birth rates in fresh IVF/ICSI cycles: a population-based nationwide registry study. Hum Reprod Open 2022;2022:hoac036. 36101708 10.1093/hropen/hoac036 PMC9464094

[hoae011-B8] Chan SY , Susarla R, Canovas D, Vasilopoulou E, Ohizua O, McCabe CJ, Hewison M, Kilby MD. Vitamin D promotes human extravillous trophoblast invasion in vitro. Placenta 2015;36:403–409. 25596923 10.1016/j.placenta.2014.12.021

[hoae011-B9] Chen C , Wang S, Zhang C, Wu X, Zhou L, Zou X, Guan T, Zhang Z, Hao J. Association between serum vitamin D level during pregnancy and recurrent spontaneous abortion: a systematic review and meta-analysis. Am J Reprod Immunol 2022;88:e13582. 35662305 10.1111/aji.13582

[hoae011-B10] Chen C , Zhai H, Cheng J, Weng P, Chen Y, Li Q, Wang C, Xia F, Wang N, Lu Y. Causal link between vitamin D and total testosterone in men: a Mendelian randomization analysis. J Clin Endocrinol Metab 2019;104:3148–3156. 30896763 10.1210/jc.2018-01874

[hoae011-B11] Dawodu A , Wagner CL. Mother–child vitamin D deficiency: an international perspective. Arch Dis Child 2007;92:737–740. 17715433 10.1136/adc.2007.122689 PMC2084036

[hoae011-B12] du Fossé NA , van der Hoorn MP, van Lith JMM, le Cessie S, Lashley EELO. Advanced paternal age is associated with an increased risk of spontaneous miscarriage: a systematic review and meta-analysis. Hum Reprod Update 2020;26:650–669. 32358607 10.1093/humupd/dmaa010 PMC7456349

[hoae011-B13] Gallagher JC , Rosen CJ. Vitamin D: 100 years of discoveries, yet controversy continues. Lancet Diabetes Endocrinol 2023;11:362–374. 37004709 10.1016/S2213-8587(23)00060-8

[hoae011-B14] Ganguly A , Tamblyn JA, Finn-Sell S, Chan S-Y, Westwood M, Gupta J, Kilby MD, Gross SR, Hewison M. Vitamin D, the placenta and early pregnancy: effects on trophoblast function. J Endocrinol 2018;236:R93–R103. 29109081 10.1530/JOE-17-0491

[hoae011-B15] Gupta P , Dabas A, Seth A, Bhatia VL, Khadgawat R, Kumar P, Balasubramanian S, Khadilkar V, Mallikarjuna HB, Godbole T et al Indian Academy of Pediatrics Revised (2021) guidelines on prevention and treatment of vitamin D deficiency and rickets. Indian Pediatr 2022;59:142–158. 34969941

[hoae011-B16] Hanley DA , Cranney A, Jones G, Whiting SJ, Leslie WD, Cole DEC, Atkinson SA, Josse RG, Feldman S, Kline GA et al; Guidelines Committee of the Scientific Advisory Council of Osteoporosis Canada. Vitamin D in adult health and disease: a review and guideline statement from Osteoporosis Canada. CMAJ 2010;182:E610–E618. 20624868 10.1503/cmaj.080663 PMC2934850

[hoae011-B17] Haq A , Wimalawansa SJ, Pludowski P, Anouti FA. Clinical practice guidelines for vitamin D in the United Arab Emirates. J Steroid Biochem Mol Biol 2018;175:4–11. 27693095 10.1016/j.jsbmb.2016.09.021

[hoae011-B18] Heyden EL , Wimalawansa SJ. Vitamin D: effects on human reproduction, pregnancy, and fetal well-being. J Steroid Biochem Mol Biol 2018;180:41–50. 29262380 10.1016/j.jsbmb.2017.12.011

[hoae011-B19] Holick MF , Binkley NC, Bischoff-Ferrari HA, Gordon CM, Hanley DA, Heaney RP, Murad MH, Weaver CM; Endocrine Society. Evaluation, treatment, and prevention of vitamin D deficiency: an Endocrine Society clinical practice guideline. J Clin Endocrinol Metab 2011;96:1911–1930. 21646368 10.1210/jc.2011-0385

[hoae011-B20] Holick MF , Chen TC. Vitamin D deficiency: a worldwide problem with health consequences1. Am J Clin Nutr 2008;87:1080S–1086S. 18400738 10.1093/ajcn/87.4.1080S

[hoae011-B21] Institute of Medicine (US) Committee to Review Dietary Reference Intakes for Vitamin D and Calcium. In: Ross AC, Taylor CL, Yaktine AL, Del Valle HB (ed). *Dietary Reference Intakes for Calcium and Vitamin D*. Washington (DC): National Academies Press (USA); 2011. Available from: https://www.ncbi.nlm.nih.gov/books/NBK56070/ (12 August 2023, date last accessed). 21796828

[hoae011-B22] Ji J , Zhai H, Zhou H, Song S, Mor G, Liao A. The role and mechanism of vitamin D-mediated regulation of Treg/Th17 balance in recurrent pregnancy loss. Am J Reprod Immunol 2019;81:e13112. 30903715 10.1111/aji.13112

[hoae011-B23] Jiang X , Ge T, Chen C-Y. The causal role of circulating vitamin D concentrations in human complex traits and diseases: a large-scale Mendelian randomization study. Sci Rep 2021;11:184. 33420236 10.1038/s41598-020-80655-w PMC7794542

[hoae011-B24] Kamat MA , Blackshaw JA, Young R, Surendran P, Burgess S, Danesh J, Butterworth AS, Staley JR. PhenoScanner V2: an expanded tool for searching human genotype-phenotype associations. Bioinformatics 2019;35:4851–4853. 31233103 10.1093/bioinformatics/btz469 PMC6853652

[hoae011-B25] Karras SN , Wagner CL, Castracane VD. Understanding vitamin D metabolism in pregnancy: from physiology to pathophysiology and clinical outcomes. Metabolism 2018;86:112–123. 29066285 10.1016/j.metabol.2017.10.001

[hoae011-B26] Kiely ME , Wagner CL, Roth DE. Vitamin D in pregnancy: where we are and where we should go. J Steroid Biochem Mol Biol 2020;201:105669. 32302652 10.1016/j.jsbmb.2020.105669

[hoae011-B27] Kim RH , Ryu BJ, Lee KM, Han JW, Lee SK. Vitamin D facilitates trophoblast invasion through induction of epithelial-mesenchymal transition. Am J Reprod Immunol 2018;79:e12796. 10.1111/aji.12796 29205625

[hoae011-B28] Kurki MI , Karjalainen J, Palta P, Sipilä TP, Kristiansson K, Donner KM, Reeve MP, Laivuori H, Aavikko M, Kaunisto MA et al; FinnGen. FinnGen provides genetic insights from a well-phenotyped isolated population. Nature 2023;613:508–518. 36653562 10.1038/s41586-022-05473-8 PMC9849126

[hoae011-B29] Li N , Wu HM, Hang F, Zhang YS, Li MJ. Women with recurrent spontaneous abortion have decreased 25(OH) vitamin D and VDR at the fetal-maternal interface. Braz J Med Biol Res 2017;50:e6527. 28902929 10.1590/1414-431X20176527 PMC5597287

[hoae011-B30] Lin S , Li J, Zhang Y, Song X, Chen G, Pei L. Maternal passive smoking, vitamin D deficiency and risk of spontaneous abortion. Nutrients2022a;14:3674. 36145050 10.3390/nu14183674 PMC9501103

[hoae011-B31] Lin S , Zhang Y, Jiang L, Li J, Chai J, Pei L, Shang X. Interactive effects of maternal vitamin D status and socio-economic status on the risk of spontaneous abortion: evidence from Henan Province, China. Nutrients2022b;14:291. 35057472 10.3390/nu14020291 PMC8780117

[hoae011-B32] Meng X , Zhang J, Wan Q, Huang J, Han T, Qu T, Yu L-L. Influence of vitamin D supplementation on reproductive outcomes of infertile patients: a systematic review and meta-analysis. Reprod Biol Endocrinol 2023;21:17. 36737817 10.1186/s12958-023-01068-8 PMC9896710

[hoae011-B33] Pereira-Santos M , Costa PRF, Assis AMO, Santos CaST, Santos DB. Obesity and vitamin D deficiency: a systematic review and meta-analysis. Obes Rev 2015;16:341–349. 25688659 10.1111/obr.12239

[hoae011-B34] Pludowski P , Holick MF, Grant WB, Konstantynowicz J, Mascarenhas MR, Haq A, Povoroznyuk V, Balatska N, Barbosa AP, Karonova T et al Vitamin D supplementation guidelines. J Steroid Biochem Mol Biol 2018;175:125–135. 28216084 10.1016/j.jsbmb.2017.01.021

[hoae011-B35] Płudowski P , Kos-Kudła B, Walczak M, Fal A, Zozulińska-Ziółkiewicz D, Sieroszewski P, Peregud-Pogorzelski J, Lauterbach R, Targowski T, Lewiński A et al Guidelines for preventing and treating vitamin D deficiency: a 2023 update in Poland. Nutrients 2023;15:695. 36771403 10.3390/nu15030695 PMC9920487

[hoae011-B36] Pourshahidi LK. Vitamin D and obesity: current perspectives and future directions. Proc Nutr Soc 2015;74:115–124. 25359323 10.1017/S0029665114001578

[hoae011-B37] Quenby S , Gallos ID, Dhillon-Smith RK, Podesek M, Stephenson MD, Fisher J, Brosens JJ, Brewin J, Ramhorst R, Lucas ES et al Miscarriage matters: the epidemiological, physical, psychological, and economic costs of early pregnancy loss. Lancet 2021;397:1658–1667. 33915094 10.1016/S0140-6736(21)00682-6

[hoae011-B38] Revez JA , Lin T, Qiao Z, Xue A, Holtz Y, Zhu Z, Zeng J, Wang H, Sidorenko J, Kemper KE et al Genome-wide association study identifies 143 loci associated with 25 hydroxyvitamin D concentration. Nat Commun 2020;11:1647. 32242144 10.1038/s41467-020-15421-7 PMC7118120

[hoae011-B39] Rostami M , Tehrani FR, Simbar M, Bidhendi Yarandi R, Minooee S, Hollis BW, Hosseinpanah F. Effectiveness of prenatal vitamin D deficiency screening and treatment program: a stratified randomized field trial. J Clin Endocrinol Metab 2018;103:2936–2948. 29788364 10.1210/jc.2018-00109

[hoae011-B40] Saraf R , Morton SMB, Camargo Jr CA, Grant CC. Global summary of maternal and newborn vitamin D status—a systematic review. Matern Child Nutr 2016;12:647–668. 26373311 10.1111/mcn.12210 PMC6860156

[hoae011-B41] Skrivankova VW , Richmond RC, Woolf BAR, Yarmolinsky J, Davies NM, Swanson SA, VanderWeele TJ, Higgins JPT, Timpson NJ, Dimou N et al Strengthening the reporting of observational studies in epidemiology using Mendelian randomization: the STROBE-MR statement. JAMA 2021;326:1614–1621. 34698778 10.1001/jama.2021.18236

[hoae011-B42] Subramanian A , Steiner AZ, Weinberg CR, Doss GL, Jukic AMZ. Preconception vitamin D and miscarriage in a prospective cohort study. Hum Reprod 2022;37:2465–2473. 35834313 10.1093/humrep/deac155 PMC9527460

[hoae011-B43] Sudlow C , Gallacher J, Allen N, Beral V, Burton P, Danesh J, Downey P, Elliott P, Green J, Landray M et al UK Biobank: an open access resource for identifying the causes of a wide range of complex diseases of middle and old age. PLoS Med 2015;12:e1001779. 25826379 10.1371/journal.pmed.1001779 PMC4380465

[hoae011-B44] Tamblyn JA , Pilarski NSP, Markland AD, Marson EJ, Devall A, Hewison M, Morris RK, Coomarasamy A. Vitamin D and miscarriage: a systematic review and meta-analysis. Fertil Steril 2022;118:111–122. 35637024 10.1016/j.fertnstert.2022.04.017

[hoae011-B45] Veleva Z , Tiitinen A, Vilska S, Hydén-Granskog C, Tomás C, Martikainen H, Tapanainen JS. High and low BMI increase the risk of miscarriage after IVF/ICSI and FET. Hum Reprod 2008;23:878–884. 18281684 10.1093/humrep/den017

[hoae011-B46] Verbanck M , Chen C-Y, Neale B, Do R. Detection of widespread horizontal pleiotropy in causal relationships inferred from Mendelian randomization between complex traits and diseases. Nat Genet 2018;50:693–698. 29686387 10.1038/s41588-018-0099-7 PMC6083837

[hoae011-B47] Wang L-Q , Yan X-T, Yan C-F, Zhang X-W, Hui L-Y, Xue M, Yu X-W. Women with recurrent miscarriage have decreased expression of 25-hydroxyvitamin D3-1α-hydroxylase by the fetal-maternal interface. PLoS One 2016;11:e0165589. 28033387 10.1371/journal.pone.0165589 PMC5199009

[hoae011-B48] WHO Guidelines Approved by the Guidelines Review Committee. Guideline: Vitamin D Supplementation in Pregnant Women. Geneva: World Health Organization, 2012. http://www.ncbi.nlm.nih.gov/books/NBK310615/ (12 August 2023, date last accessed).

[hoae011-B49] Xu Q-H , Muyayalo KP, Zhang Y-J, Wang H, Lin X-X, Liao A-H. Altered vitamin D metabolism is involved in the dysregulation of γδT cell function and their crosstalk with trophoblasts in recurrent pregnancy loss. Am J Reprod Immunol 2023;89:e13581. 35704547 10.1111/aji.13581

[hoae011-B50] Yan X , Wang L, Yan C, Zhang X, Hui L, Sheng Q, Xue M, Yu X. Decreased expression of the vitamin D receptor in women with recurrent pregnancy loss. Arch Biochem Biophys 2016;606:128–133. 27477959 10.1016/j.abb.2016.07.021

[hoae011-B51] Zhang F , Wang Z, Lian R, Diao L, Li Y, Wu Y, Yin T, Huang C. Intrauterine perfusion of dexamethasone improves pregnancy outcomes in recurrent reproductive failure patients with elevated uterine natural killer cells. a retrospective cohort study. Am J Reprod Immunol2023a;90:e13796. 38009055 10.1111/aji.13796

[hoae011-B52] Zhang H , Wang S, Tuo L, Zhai Q, Cui J, Chen D, Xu D. Relationship between maternal vitamin D levels and adverse outcomes. Nutrients 2022;14:4230. 36296914 10.3390/nu14204230 PMC9610169

[hoae011-B53] Zhang JY , Wu P, Chen D, Ning F, Lu Q, Qiu X, Hewison M, Tamblyn JA, Kilby MD, Lash GE. Vitamin D promotes trophoblast cell induced separation of vascular smooth muscle cells in vascular remodeling via induction of G-CSF. Front Cell Dev Biol 2020;8:601043. 33415106 10.3389/fcell.2020.601043 PMC7783206

[hoae011-B54] Zhang N , Liao Y, Zhao H, Chen T, Jia F, Yu Y, Zhu S, Wang C, Zhang W, Liu X. Polycystic ovary syndrome and 25-hydroxyvitamin D: a bidirectional two-sample Mendelian randomization study. Front Endocrinol (Lausanne) 2023b;14:1110341. 36967791 10.3389/fendo.2023.1110341 PMC10034407

[hoae011-B55] Zhou A , Hyppönen E. Vitamin D deficiency and C-reactive protein: a bidirectional Mendelian randomization study. Int J Epidemiol 2023;52:260–271. 35579027 10.1093/ije/dyac087 PMC9908047

[hoae011-B56] Zhou X , Wu X, Luo X, Shao J, Guo D, Deng B, Wu Z. Effect of vitamin D supplementation on in vitro fertilization outcomes: a trial sequential meta-analysis of 5 randomized controlled trials. Front Endocrinol (Lausanne) 2022;13:852428. 35370977 10.3389/fendo.2022.852428 PMC8969598

