# Supplementary material for: No evidence of a causal relationship between miscarriage and 25-hydroxyvitamin D: a Mendelian randomization study
Source: Hum Reprod Open. 2024 Feb 19;2024(2):hoae011. doi: 10.1093/hropen/hoae011 (PMC10918637; doi:10.1093/hropen/hoae011)
Supplement: hoae011_Supplementary_Data [file hoae011_supplementary_data.zip › Supplementary Table.pdf]

Supplementary Table S1. Details of exposures (serum 25-hydroxyvitamin D concentration and vitamin D deficiency) and outcomes (miscarriage and number of miscarriages) included in the Mendelian randomization study.

Trait	Number of cases	Number of controls	Sample size	Number of SNPs	Data source	Population	Gender	Year	PubMed ID	First author	Download link
Serum 25-hydroxyvitamin D concentration	NA	NA	417580	8806780	UK Biobank	European	Males and Females	2020	32242144*	Joana A. Revez	https://cnsgenomics.com/data/revez_20/Revezetal2020_25OHD_BMIcov.gz
Vitamin D deficiency	426	354812	355238	20169720	FinnGen	European	Males and Females	2023	NA	NA	https://storage.googleapis.com/finngen-public-data-r9/summary_stats/finngen_R9_E4_VIT_D_DEF.gz
Miscarriage	16906	149622	166528	20155736	FinnGen	European	Females	2023	NA	NA	https://storage.googleapis.com/finngen-public-data-r9/summary_stats/finngen_R9_O15_ABORT_SPONTAN.gz
Number of miscarriages	NA	NA	78700	9851867	MRC-IEU	European	Females	2018	NA	Ben Elsworth	https://gwas.mrcieu.ac.uk/datasets/ukb-b-419/

* Revez JA, Lin T, Qiao Z, Xue A, Holtz Y, Zhu Z, Zeng J, Wang H, Sidorenko J, Kemper KE, et al. Genome-wide association study identifies 143 loci associated with 25 hydroxyvitamin D concentration. Nat Commun 2020;11:1647.

Supplementary Table S2. Genetic instrumental variables utilized in the Mendelian randomization analysis of serum 25-hydroxyvitamin D concentration on the odds and number of miscarriages.

SNPs	Chr	Position	EA	OA	EAF	R^2	F	Association with 25OHD			Association with miscarriage			Association with No. of miscarriages		
								Beta	SE	P-value	Beta	SE	P-value	Beta	SE	P-value
rs1047891	2	211540507	C	A	0.684	0.0000681	28.346	0.013	0.002	2.6E-09	-0.016	0.012	0.203	-0.003	0.004	0.370
rs10822145	10	64934548	C	T	0.525	0.0000789	32.714	0.013	0.002	1.6E-10	0.017	0.012	0.142	0.002	0.004	0.510
rs10859995	12	96375682	T	C	0.417	0.0007761	323.287	0.040	0.002	1.1E-89	-0.017	0.012	0.162	-0.002	0.004	0.660
rs10908469	1	155468732	A	C	0.730	0.0000995	41.231	-0.016	0.002	6.7E-13	-0.004	0.013	0.755	0.007	0.004	0.063
rs11023212	11	14431709	G	A	0.656	0.0023904	975.109	0.073	0.002	1E-200	0.015	0.012	0.222	-0.008	0.004	0.034
rs11076175	16	57006378	A	G	0.822	0.0001685	69.910	-0.024	0.003	9.5E-21	-0.009	0.015	0.560	-0.004	0.005	0.380
rs11122455*	1	230304930	C	G	0.386	0.0000759	31.576	-0.013	0.002	3.4E-10	0.010	0.012	0.379	-0.007	0.004	0.071
rs11182428	12	38526387	T	C	0.480	0.0000761	31.639	0.012	0.002	3.2E-10	0.008	0.012	0.464	-0.005	0.004	0.170
rs1149608	11	76494928	C	T	0.825	0.0001332	54.858	-0.021	0.003	1.3E-16	-0.018	0.016	0.258	-0.003	0.005	0.520
rs11542462	16	82033810	G	A	0.866	0.0001258	52.353	0.023	0.003	5.8E-16	0.024	0.020	0.220	-0.002	0.005	0.770
rs11606*#	19	54658102	C	G	0.575	0.0000615	24.685	-0.011	0.002	2.8E-08	0.014	0.012	0.235	0.002	0.004	0.650
rs11751024	6	32586236	C	A	0.603	0.0000756	31.379	0.013	0.002	3.8E-10	0.003	0.012	0.818	0.000	0.004	0.970
rs117592720	11	14980137	T	C	0.974	0.0002591	106.697	-0.071	0.006	7.2E-31	0.075	0.030	0.013	0.008	0.011	0.490
rs117862422	11	13210063	T	C	0.986	0.0000786	32.467	0.054	0.009	1.9E-10	-0.025	0.029	0.374	0.011	0.015	0.470
rs12056768	8	116988527	T	G	0.417	0.0002343	96.877	0.022	0.002	3.3E-28	-0.010	0.012	0.386	-0.004	0.004	0.270
rs12123821	1	152179152	C	T	0.952	0.0005401	224.956	-0.077	0.005	3.7E-63	-0.031	0.030	0.292	-0.011	0.008	0.200
rs12317268	12	21352541	A	G	0.849	0.0001091	45.205	0.021	0.003	5.3E-14	0.003	0.013	0.825	0.003	0.005	0.500
rs12462826	19	11955767	G	A	0.631	0.0000801	32.861	0.013	0.002	1.5E-10	0.007	0.012	0.587	NA	NA	NA
rs1260326	2	27730940	T	C	0.393	0.0002408	100.274	-0.022	0.002	4E-29	-0.017	0.012	0.154	0.002	0.004	0.650
rs12949853	17	7570878	G	A	0.193	0.000065	25.948	-0.014	0.003	1.2E-08	-0.007	0.015	0.664	0.009	0.005	0.048
rs13011615	2	21274167	A	T	0.865	0.0000672	27.603	-0.017	0.003	4E-09	-0.015	0.017	0.364	0.002	0.005	0.740
rs13284054	9	107669073	T	C	0.882	0.0000715	28.897	-0.019	0.003	1.8E-09	0.007	0.019	0.727	-0.002	0.006	0.700
rs142004400	14	50829560	A	C	0.965	0.0000737	30.492	0.033	0.005	6.4E-10	-0.109	0.040	0.006	0.000	0.010	0.970
rs142158911	19	11190534	G	A	0.885	0.0001444	59.441	-0.027	0.003	7.2E-18	0.023	0.019	0.229	0.006	0.006	0.300
rs143069752	6	40962537	T	A	0.933	0.0000631	25.981	-0.022	0.004	1.2E-08	0.012	0.024	0.633	0.011	0.007	0.110
rs143488652	11	13508611	A	G	0.987	0.0001047	43.313	0.063	0.009	1.8E-13	-0.111	0.123	0.367	0.013	0.015	0.390
rs146128209	11	14683683	A	G	0.929	0.0004322	179.069	0.057	0.004	1.4E-50	0.013	0.028	0.653	0.007	0.007	0.350
rs1532085	15	58683366	A	G	0.385	0.0003206	133.479	-0.026	0.002	3.6E-38	-0.013	0.012	0.279	0.002	0.004	0.540
rs17144574	7	21563471	T	C	0.766	0.0000834	34.029	0.015	0.002	6.7E-11	0.010	0.014	0.475	0.005	0.004	0.250
rs1792287	11	71057911	A	G	0.735	0.0001917	78.929	-0.022	0.002	2.7E-23	-0.011	0.013	0.410	0.002	0.004	0.650
rs1800588	15	58723675	C	T	0.785	0.0003678	153.138	0.033	0.002	1.5E-43	-0.001	0.013	0.963	0.005	0.004	0.260
rs1858889	7	107117447	A	C	0.498	0.000062	25.768	-0.011	0.002	1.4E-08	0.001	0.012	0.948	-0.001	0.004	0.740
rs189407772	4	100146674	A	G	0.977	0.000125	51.562	-0.053	0.007	1E-15	-0.021	0.082	0.796	-0.013	0.012	0.280
rs1966478	5	118627319	T	C	0.307	0.0000649	26.860	0.012	0.002	6.9E-09	0.001	0.012	0.949	-0.004	0.004	0.340
rs2012736	2	234622379	C	A	0.919	0.0003452	142.879	0.048	0.004	1.1E-40	0.041	0.022	0.063	0.007	0.007	0.260
rs2037511	18	61366207	G	A	0.834	0.0000804	33.448	-0.017	0.003	1.1E-10	0.018	0.016	0.261	-0.003	0.005	0.560
rs2074735	22	31535872	G	C	0.936	0.0000927	38.598	-0.028	0.004	4E-12	-0.011	0.017	0.511	0.000	0.007	0.950
rs212100	19	48376995	T	C	0.164	0.0011837	491.909	0.066	0.003	8E-136	-0.005	0.016	0.740	0.013	0.005	0.010
rs2123930	15	100231033	G	A	0.721	0.0000802	33.062	0.014	0.002	1.3E-10	0.010	0.012	0.424	-0.003	0.004	0.400
rs2131925	1	63025942	G	T	0.356	0.0002267	94.403	0.022	0.002	2.4E-27	-0.002	0.013	0.869	0.003	0.004	0.430
rs2207132	20	39142516	G	A	0.967	0.000095	39.551	0.039	0.005	2E-12	0.000	0.024	0.985	0.004	0.010	0.650
rs2229742	21	16339172	G	C	0.897	0.0001172	48.799	0.025	0.003	5.9E-15	0.022	0.018	0.234	-0.003	0.006	0.600
rs2246832*#	3	49881134	A	T	0.499	0.000148	61.444	-0.017	0.002	1.9E-18	-0.001	0.012	0.912	0.002	0.004	0.500
rs2297991	10	113913222	T	C	0.279	0.0000583	24.286	-0.012	0.002	3.5E-08	0.002	0.012	0.882	-0.003	0.004	0.400
rs2346264	7	133536351	A	C	0.217	0.0000735	30.053	0.015	0.002	8.9E-10	-0.012	0.016	0.446	-0.002	0.004	0.690
rs2608984	6	131918839	A	T	0.835	0.0001201	49.913	0.021	0.003	2.8E-15	0.008	0.014	0.558	-0.004	0.005	0.370
rs2642439	1	220970499	A	G	0.315	0.000091	37.782	0.015	0.002	6.2E-12	-0.011	0.013	0.411	0.007	0.004	0.070
rs2659007	17	79217478	G	A	0.549	0.0000645	26.347	-0.011	0.002	9.8E-09	0.006	0.012	0.579	-0.002	0.004	0.670
rs2756119	14	104001517	G	A	0.617	0.0000702	28.532	-0.012	0.002	2.4E-09	-0.026	0.012	0.030	-0.002	0.004	0.550
rs2847500	11	120114421	G	A	0.876	0.0001058	44.034	0.022	0.003	1.2E-13	-0.023	0.016	0.165	-0.002	0.005	0.700
rs2952289	17	66464414	C	T	0.202	0.000103	42.409	-0.018	0.002	3.2E-13	0.015	0.015	0.322	-0.002	0.004	0.620
rs34177108	16	89893375	C	A	0.732	0.0000592	24.081	0.012	0.002	4E-08	0.002	0.014	0.880	0.004	0.004	0.370
rs34186890	3	141720712	A	G	0.740	0.0000864	35.818	0.015	0.002	2.3E-11	-0.011	0.013	0.432	-0.001	0.004	0.730
rs34284484	13	55710231	T	G	0.714	0.0000619	25.669	0.012	0.002	1.4E-08	-0.001	0.013	0.964	-0.006	0.004	0.120
rs34726834	8	25889606	C	T	0.748	0.0000844	34.716	-0.015	0.002	4.5E-11	0.001	0.013	0.968	-0.007	0.004	0.073
rs35408430	1	17560195	C	T	0.658	0.0002049	85.042	0.021	0.002	5.8E-25	0.021	0.012	0.080	0.009	0.004	0.012
rs3814995	19	36342212	C	T	0.688	0.0000686	28.543	0.013	0.002	2.3E-09	-0.002	0.012	0.874	0.003	0.004	0.450
rs3890624	4	166251068	A	G	0.605	0.0000608	24.466	-0.011	0.002	3.2E-08	0.006	0.012	0.605	0.007	0.004	0.049
rs4121823	18	47144223	T	A	0.155	0.0000895	36.661	0.019	0.003	1.3E-11	-0.007	0.016	0.659	0.006	0.005	0.240
rs41301394	7	75612803	C	T	0.718	0.0000644	26.761	-0.013	0.002	7.1E-09	0.013	0.012	0.291	0.006	0.004	0.110
rs429358	19	45411941	T	C	0.848	0.0001391	57.907	0.023	0.003	1.8E-17	-0.008	0.015	0.609	0.000	0.005	0.980
rs4306928	4	72367671	T	C	0.168	0.0001753	72.718	-0.025	0.003	1.6E-21	0.020	0.016	0.194	0.001	0.005	0.790
rs4364259	4	15892159	G	A	0.798	0.0000879	35.790	-0.017	0.002	2.2E-11	0.031	0.014	0.025	0.005	0.004	0.310
rs4418728	10	94839724	G	T	0.548	0.0000728	30.257	-0.012	0.002	7.5E-10	0.014	0.012	0.217	0.001	0.004	0.870
rs4616820	4	57745481	C	T	0.535	0.0000757	30.878	0.012	0.002	5.3E-10	0.011	0.012	0.356	-0.006	0.004	0.099
rs4694423	4	72554159	C	A	0.584	0.0049458	2063.428	0.101	0.002	1E-200	-0.014	0.012	0.233	-0.009	0.004	0.018
rs512083	1	46027355	T	C	0.539	0.000074	30.513	-0.012	0.002	6.6E-10	-0.018	0.012	0.110	-0.001	0.004	0.740
rs57459725	8	61312205	C	G	0.867	0.0000719	29.799	0.018	0.003	1.1E-09	0.025	0.015	0.095	0.003	0.005	0.560
rs58387006	2	32579999	A	C	0.778	0.000065	26.889	0.014	0.002	6.3E-09	-0.006	0.014	0.686	-0.005	0.004	0.230
rs6003465	22	23365501	T	C	0.668	0.000063	25.933	0.012	0.002	1.2E-08	0.008	0.013	0.556	0.001	0.004	0.770
rs61815559	1	152271219	A	T	0.969	0.0004352	179.228	-0.085	0.006	9.6E-51	-0.040	0.066	0.543	-0.009	0.010	0.400
rs62012766	15	63852834	T	C	0.843	0.0000873	36.300	0.018	0.003	1.6E-11	-0.025	0.014	0.067	-0.010	0.005	0.040
rs62115743	19	51514874	C	T	0.918	0.0001128	46.108	-0.027	0.004	3.2E-14	0.012	0.019	0.520	-0.006	0.006	0.390
rs6438900	3	125148287	C	G	0.742	0.0000682	27.941	-0.013	0.002	3.4E-09	-0.016	0.013	0.215	-0.002	0.004	0.670
rs6547409	2	21190209	C	T	0.950	0.0000738	30.484	-0.028	0.005	6.6E-10	-0.015	0.022	0.492	0.001	0.008	0.910
rs6671730	1	2339139	G	A	0.566	0.0001123	46.489	0.015	0.002	2.3E-14	0.005	0.012	0.659	-0.001	0.004	0.690
rs6723486	2	58979783	C	T	0.389	0.0000588	24.018	0.011	0.002	4.2E-08	0.010	0.012	0.368	-0.004	0.004	0.300
rs6724965	2	101440151	A	G	0.829	0.0000781	32.523	0.017	0.003	1.8E-10	-0.018	0.014	0.213	0.006	0.005	0.190
rs6834488	4	88178919	C	T	0.576	0.0001046	43.252	0.015	0.002	2E-13	0.006	0.012	0.603	0.004	0.004	0.250
rs71467497	11	70471414	T	C	0.971	0.0000618	25.430	0.033	0.006	1.7E-08	-0.020	0.035	0.559	0.022	0.011	0.039
rs71601787	4	72866015	G	A	0.673	0.0007734	316.713	-0.042	0.002	6.1E-88	-0.015	0.013	0.225	0.002	0.004	0.660
rs72834856	6	22801858	T	G	0.928	0.0000745	30.876	0.024	0.004	4.9E-10	-0.006	0.024	0.802	0.011	0.007	0.110
rs73413596	12	111582630	T	C	0.926	0.0000772	31.846	-0.024	0.004	2.9E-10	-0.042	0.025	0.090	0.006	0.007	0.400
rs7439366	4	69964338	T	C	0.544	0.0004945	205.932	0.032	0.002	5.2E-58	-0.018	0.012	0.132	0.002	0.004	0.540
rs7522116	1	41835685	C	T	0.434	0.0000781	32.041	0.013	0.002	2.6E-10	0.000	0.012	0.983	NA	NA	NA

rs7528419	1	109817192	A	G	0.775	0.0001404	58.453	-0.020	0.002	1.3E-17	-0.020	0.014	0.165	0.000	0.004	0.990
rs7569755	2	118648261	G	A	0.709	0.0000723	29.542	-0.013	0.002	1.2E-09	-0.001	0.014	0.929	0.003	0.004	0.510
rs7657132	4	73416601	A	G	0.683	0.0000858	34.669	0.014	0.002	4.4E-11	-0.004	0.012	0.734	0.004	0.004	0.330
rs77924615	16	20392332	G	A	0.807	0.0000655	26.619	0.014	0.003	8.1E-09	-0.013	0.014	0.358	-0.002	0.005	0.680
rs78649910	4	3482213	T	A	0.894	0.0000709	29.064	0.019	0.003	1.6E-09	0.014	0.018	0.417	0.001	0.006	0.810
rs79598313	1	27284913	C	T	0.977	0.0000626	26.029	0.037	0.006	1.1E-08	-0.036	0.031	0.243	-0.016	0.012	0.170
rs79687284	1	214150821	G	C	0.965	0.0000582	24.222	0.030	0.005	3.8E-08	-0.043	0.044	0.328	-0.010	0.010	0.320
rs8018720	14	39556185	G	C	0.177	0.0004282	178.313	0.038	0.003	2E-50	-0.013	0.016	0.438	0.000	0.005	0.940
rs804281	8	11611865	A	G	0.416	0.0001206	50.081	-0.016	0.002	2.5E-15	-0.023	0.012	0.068	-0.005	0.004	0.160
rs8063565	16	30883965	G	C	0.266	0.0000594	24.691	-0.012	0.002	2.7E-08	-0.017	0.013	0.180	0.000	0.004	0.930
rs8091117	18	28919794	C	A	0.935	0.0000771	32.100	0.025	0.004	2.3E-10	-0.024	0.022	0.268	0.000	0.007	0.970
rs8107974	19	19388500	A	T	0.924	0.0002101	87.355	-0.039	0.004	1.5E-25	-0.002	0.023	0.947	0.001	0.007	0.920
rs8121940	20	52742306	C	G	0.805	0.000447	185.826	0.038	0.002	1.7E-52	0.001	0.014	0.922	-0.004	0.005	0.420
rs8181687	12	68661596	G	A	0.421	0.0000667	27.631	-0.012	0.002	4E-09	-0.025	0.012	0.030	-0.001	0.004	0.710
rs9409266	9	125745042	G	A	0.139	0.000081	33.634	0.018	0.003	8.7E-11	0.003	0.015	0.846	NA	NA	NA
rs9467550	6	25653401	A	G	0.895	0.0000672	27.845	0.019	0.003	3.4E-09	-0.002	0.018	0.912	0.010	0.006	0.098
rs9476310	6	57767576	C	T	0.489	0.0000687	28.404	-0.012	0.002	2.7E-09	0.011	0.012	0.335	-0.003	0.004	0.430
rs964184	11	116648917	G	C	0.132	0.0004264	177.560	-0.043	0.003	3.4E-50	0.011	0.016	0.494	-0.010	0.005	0.068

Abbreviations: SNPs, single nucleotide polymorphism; Chr, Chromosome; Pos, position; EA, effect allele; OA, other allele; EAF, effect allele frequency; R^2, proportion of variance explained by SNP; SE, standard error; 25OHD, serum 25-Hydroxyvitamin D levels; No. of miscarriages, number of miscarriages. NA means that the corresponding SNPs cannot be extracted in GWAS summary data of outcomes.

* and # indicate that the corresponding SNPs were eliminated for being palindromic with intermediate allele frequencies.

Supplementary Table S3. Genetic instrumental variables utilized in the Mendelian randomization analysis of vitamin D deficiency on the odds and number of miscarriages.

SNPs	Chr	Position	EA	OA	EAF	R^2	F	Association with VDD			Association with miscarriage			Association with No. of miscarriages		
								Beta	SE	P-value	Beta	SE	P-value	Beta	SE	P-value
rs113048353	20	37293687	C	T	0.033	0.031	11473.574	0.701	0.149	2.69E-06	0.009	0.033	0.785	0.008	0.015	0.630
rs141054898	10	91910865	G	A	0.003	0.017	6226.2573	1.660	0.334	6.6E-07	-0.233	0.103	0.024	0.011	0.015	0.430
rs149704870	18	14338017	C	A	0.253	0.054	20121.894	-0.377	0.082	4.07E-06	0.000	0.013	0.985	NA	NA	NA
rs16864904	3	189958368	T	C	0.074	0.067	25700.364	-0.702	0.154	4.89E-06	-0.013	0.022	0.549	-0.001	0.007	0.870
rs36120117	4	89246482	T	C	0.137	0.070	26856.283	-0.546	0.111	8.02E-07	0.004	0.017	0.835	0.001	0.006	0.840
rs6466644	7	118103262	C	T	0.767	0.056	20882.408	0.394	0.085	3.7E-06	-0.012	0.014	0.397	NA	NA	NA
rs9905991	17	82094197	G	A	0.534	0.050	18873.48	-0.318	0.066	1.51E-06	-0.003	0.012	0.789	0.003	0.004	0.470

Abbreviations: SNPs, single nucleotide polymorphism; Chr, Chromosome; Pos, position; EA, effect allele; OA, other allele; EAF, effect allele frequency; R^2, proportion of variance explained by SNP; SE, standard error; VDD, Vitamin D deficiency; No. of miscarriages, number of miscarriages.
NA means that the corresponding SNPs cannot be extracted in GWAS summary data of outcomes.
