# Supplementary material for: No evidence of a causal relationship between miscarriage and 25-hydroxyvitamin D: a Mendelian randomization study
Source: Hum Reprod Open. 2024 Feb 19;2024(2):hoae011. doi: 10.1093/hropen/hoae011 (PMC10918637; doi:10.1093/hropen/hoae011)
Supplement: hoae011_Supplementary_Data [file hoae011_supplementary_data.zip › Supplementary Figure.pdf]

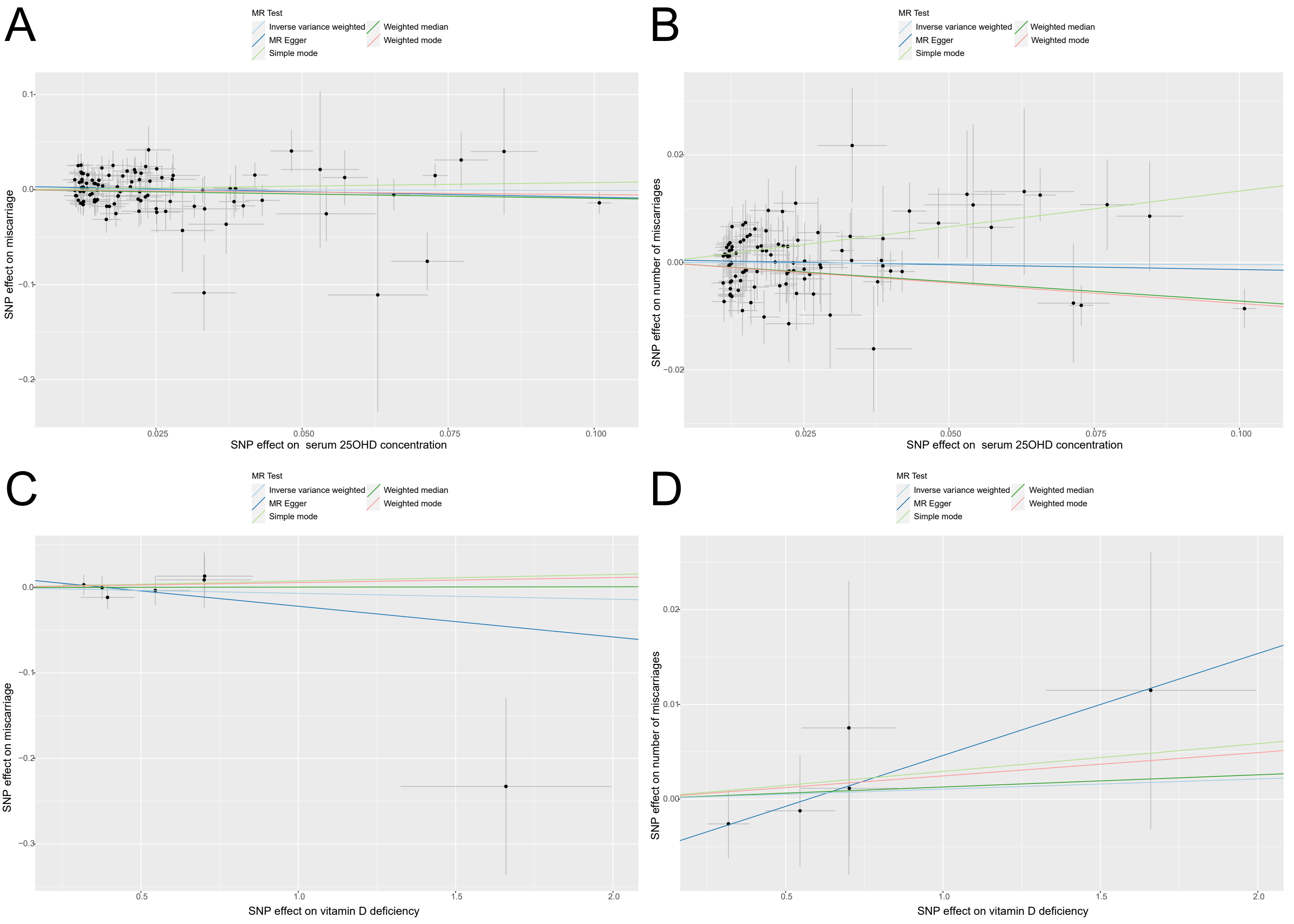

Supplementary Figure S1 Scatter plots for the effect of serum 25OHD concentration on the odds (A) and number of miscarriages (B). Scatter plots for the effect of vitamin D deficiency on the odds (C) and number of miscarriages (D).

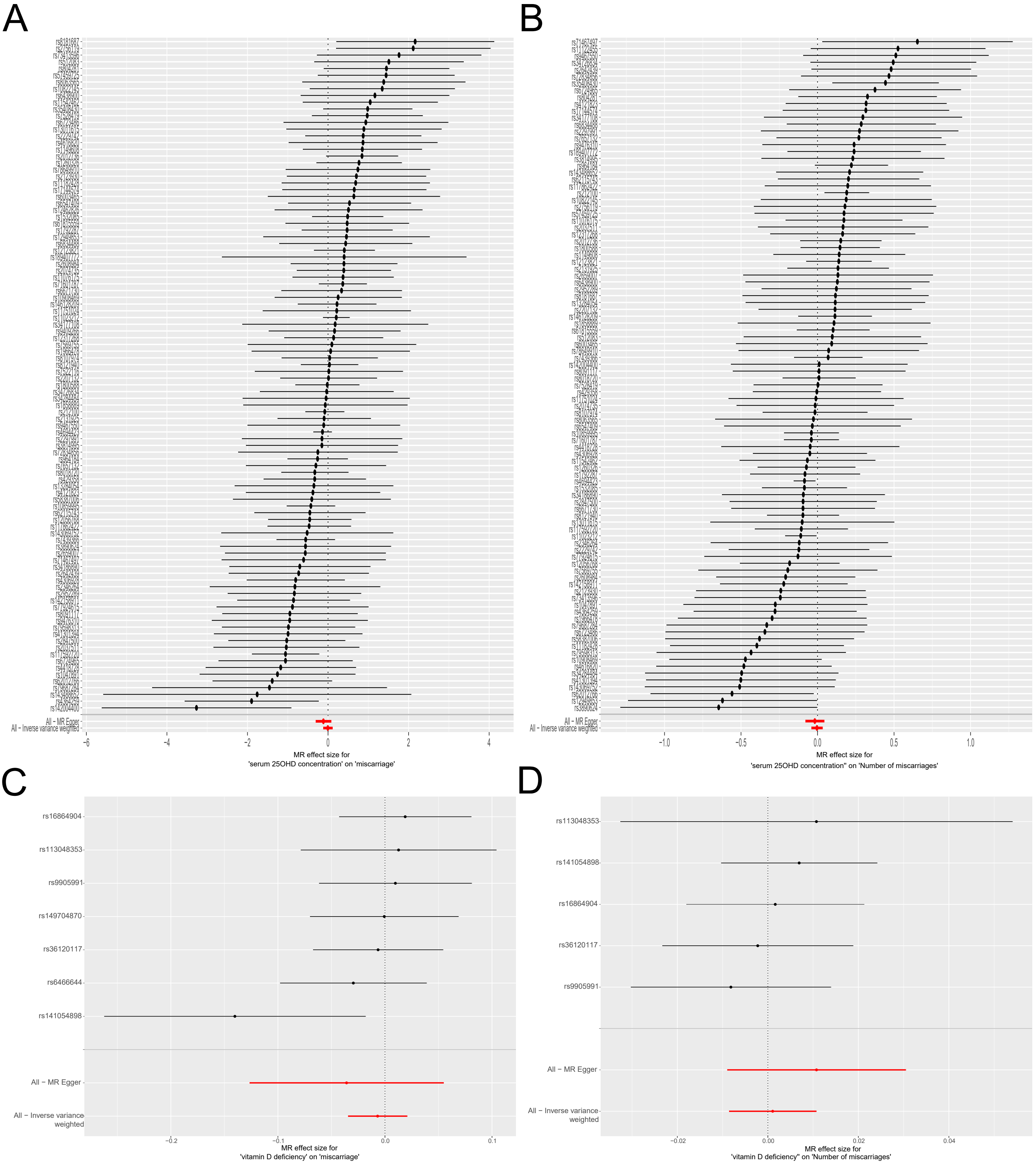

Supplementary Figure S2 Forest plots of the association of serum 25OHD concentration with the odds (A) and number of miscarriages (B). Forest plots of the association of vitamin D deficiency on the odds (C) and number of miscarriages (D). All effect sizes in the above graph are expressed as beta.

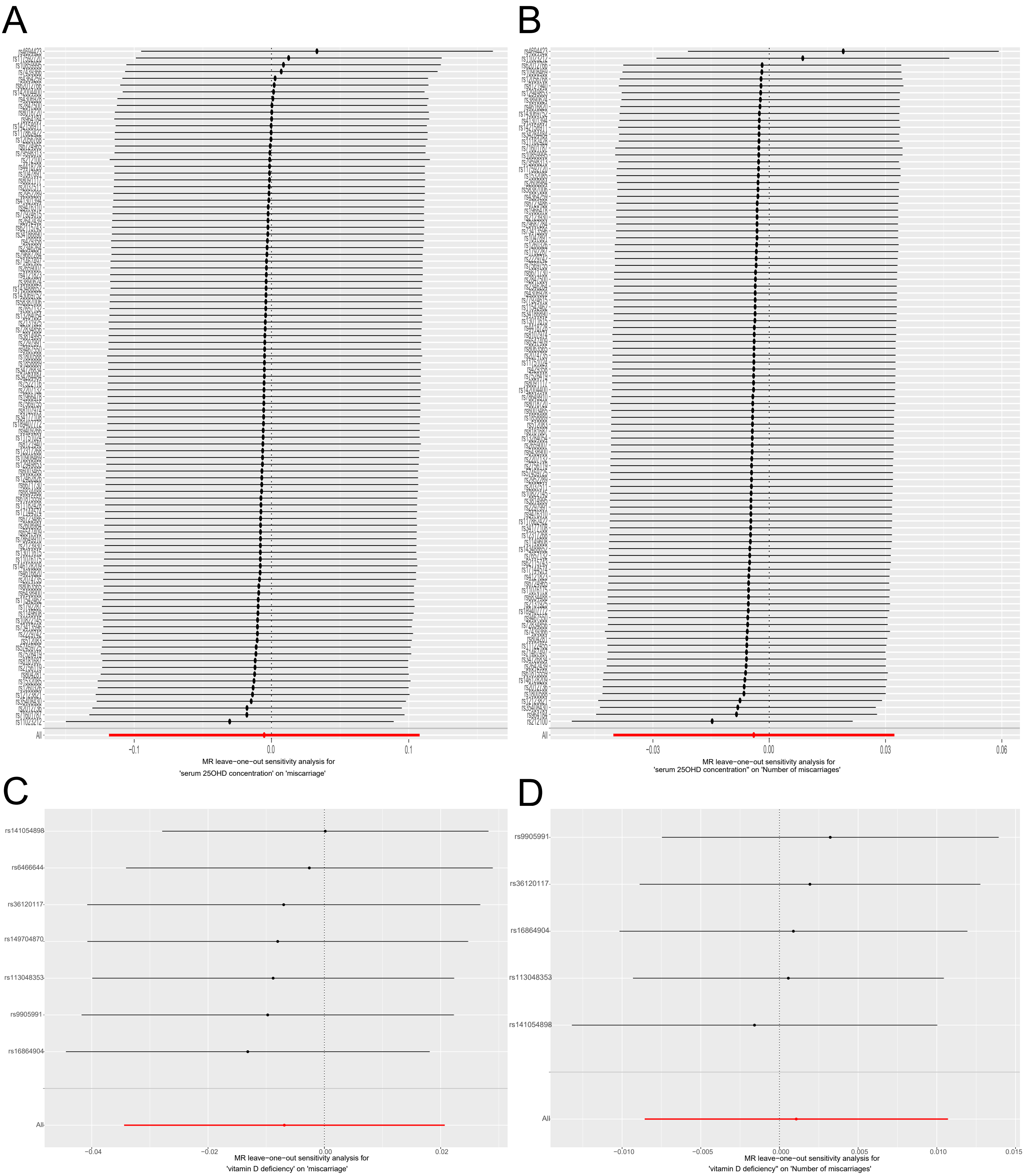

Supplementary Figure S3 MR leave-one-out sensitivity analysis for the effect of serum 25OHD concentration on the odds (A) and number of miscarriages (B). MR leave-one-out sensitivity analysis for the effect of vitamin D deficiency on the odds (C) and number of miscarriages (D). All effect sizes in the above graph are expressed as beta.

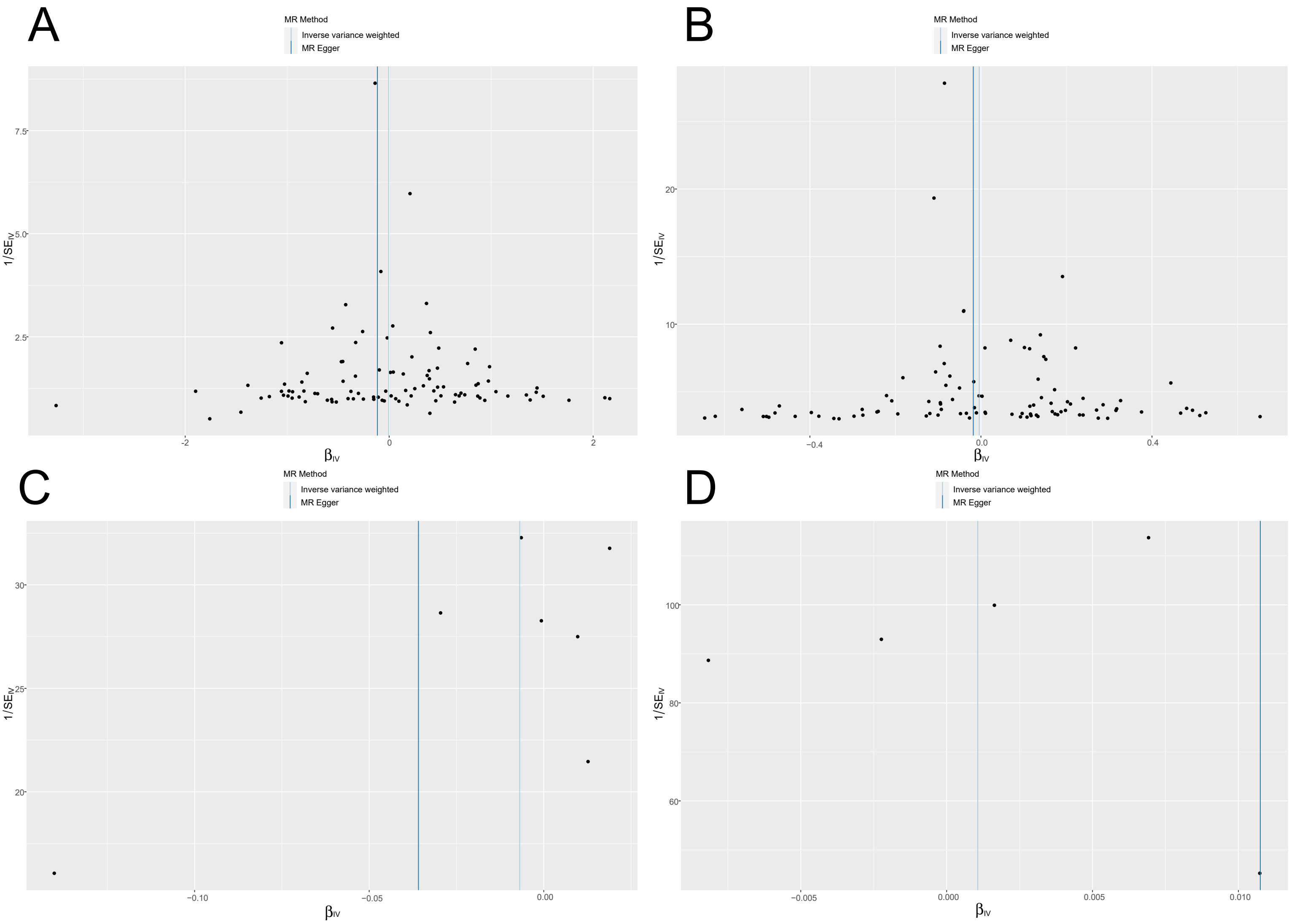

Supplementary Figure S4 Funnel plots for the effect of serum 25OHD concentration on the odds (A) and number of miscarriages (B). Funnel plots for the effect of vitamin D deficiency on the odds (C) and number of miscarriages (D).
